# Evaluation of the Mercy weight estimation method in Ouelessebougou, Mali

**DOI:** 10.1186/1471-2458-14-270

**Published:** 2014-03-21

**Authors:** Alassane Dicko, Mohamed Lamine Alhousseini, Bouran Sidibé, Moussa Traoré, Susan M Abdel-Rahman

**Affiliations:** 1 Department of Public Health & Malaria Research and Training Center, Faculty of Medicine, Pharmacy and Dentistry, University of Bamako, P.O. Box 1805 Bamako, Mali; 2 Division of Clinical Pharmacology and Medical Toxicology, Children’s Mercy Hospital, 2401 Gillham Rd., Suite 041, Kansas, MO 64108, USA

## Abstract

**Background:**

This study evaluated the performance of a new weight estimation strategy (Mercy Method) with four existing weight-estimation methods (APLS, ARC, Broselow, and Nelson) in children from Ouelessebougou, Mali.

**Methods:**

Otherwise healthy children, 2 mos to 16 yrs, were enrolled and weight, height, humeral length (HL) and mid-upper arm circumference (MUAC) obtained by trained raters. Weight estimation was performed as described for each method. Predicted weights were regressed against actual weights. Agreement between estimated and actual weight was determined using Bland-Altman plots with log-transformation. Predictive performance of each method was assessed using residual error (RE), percentage error (PE), root mean square error (RMSE), and percent predicted within 10, 20 and 30% of actual weight.

**Results:**

473 children (8.1 ± 4.8 yr, 25.1 ± 14.5 kg, 120.9 ± 29.5 cm) participated in this study. The Mercy Method (MM) offered the best correlation between actual and estimated weight when compared with the other methods (r^2^ = 0.97 vs. 0.80-0.94). The MM also demonstrated the lowest ME (0.06 vs. 0.92-4.1 kg), MPE (1.6 vs. 7.8-19.8%) and RMSE (2.6 vs. 3.0-6.7). Finally, the MM estimated weight within 20% of actual for nearly all children (97%) as opposed to the other methods for which these values ranged from 50-69%.

**Conclusions:**

The MM performed extremely well in Malian children with performance characteristics comparable to those observed for U.S and India and could be used in sub-Saharan African children without modification extending the utility of this weight estimation strategy.

## Background

In pediatric medicine, the provision of basic health services necessitates knowledge of the child’s weight. Weight is essential for evaluating normal growth and development, examining the adequacy of nutrition, and delivering proper medication doses when children fall ill. Accurately assessing weight is especially important in African settings where an estimated one in four children are nutritionally compromised [1-3]. Sadly, a substantial fraction of children in West and Central Africa do not have access to health care, and the vast majority of facilities to which these children have access do not have functional, calibrated, scales that demonstrate the precision necessary to accurately determine weight [4-9]. Consequently, weight estimation is relied upon heavily as a surrogate for scale-derived weights.

Though there exist numerous published strategies for pediatric weight estimation, they remain less than ideal for use in African settings. Most methods were developed using Western standards for growth and development and few have been validated for use in African populations [10-15]. Even when applied to the population in whom they were defined, the predictive power of these methods falls short. Most fail to account for the extremes of body composition and stature that are observed in children of the same age, some require multiple or complex formulae, others require subjective determinations of habitus, and essentially all of them have restrictions on the age or length of children for whom they are designed [15-20].

In response to the lack of a robust, broadly-applicable weight estimation method for children investigators recently developed the Mercy Method [21]. It is an anthropometric-based method that predicts weight in children 2 months through 16 years of age. It incorporates surrogates of both height and girth which prove to be more accurate estimates relying on a single variable. It is not based on age which may be unavailable in countries where birth records are not maintained and does not require total body length which may be difficult to obtain in uncooperative children or those being swaddled by a caregiver. The Mercy Method was shown to be highly accurate irrespective of nutritional status and predicts weight within 20% of actual in 98% of U.S. children [21]. A recent validation study in South Asia demonstrated that this method performed exceptionally well in children from India [22]. This study was undertaken to examine the performance of the Mercy Method in a West African pediatric population.

## Methods

### Subjects and study design

This was a prospective, single-center study conducted in July- August 2011, in Mali. Otherwise healthy children 2 months through 16 years of age living in the village of Ouelessebougou were eligible for participation. The study was explained and advertised during meeting with the community leaders and health workers in Ouelessebougou. Parents were asked to bring their children to the research center and to provide individual informed consent. All the children presenting to the research center and for whom informed consent and assent (7–17 years) were obtained were enrolled unless they presented with any of the following exclusion criteria: 1) known or apparent limb deformities, 2) unable to be positioned for height/length measurements, 3) underlying pathological condition that would produce abnormal body composition for age (e.g. severe edema). To ensure that an appropriate cross-section of children was sampled, participants were stratified by age in one year age brackets (e.g. 0–1 years, 1–2 years…16-17 years) prior to enrollment. All children were enrolled with written informed permission, and assent where appropriate, under a protocol that was reviewed and approved by the University of Bamako Ethics Committee (EC) and the Ethics Committee of the World Health Organization, Geneva. Since the local language of Ouelessebougou is a spoken language, for parents and children who cannot read and understand French, the permission/assent forms were verbally translated into the local language from the EC approved French language document in presence of an independent witness.

### Data collection

Children came to study site on the day of their participation where anthopometric measurements including; height, weight, humeral length and mid-upper arm circumference were performed by one of four trained raters. Children able to stand unassisted were positioned with their heels, buttocks, and head in contact with the height rule of a portable stadiometer. The head was aligned in the Frankfort horizontal plane and the head piece of the stadiometer lowered making very effort to compress the hair prior to recording the child’s height. In infants unable to stand, recumbent length was measured using an infantometer. The child was placed on an examining table, the head oriented in the Frankfort plane, and gentle pressure applied at the knees to keep the legs straight before marking the length. Each participant was weighed in there undergarments or other light weight clothing using a portable scale that was calibrated daily. Humeral Length (HL) was measured from the most upper edge of the posterior border of the acromion process, down the posterior surface of the arm, to the tip of the olecranon process. Mid-upper arm circumference (MUAC) was measured at the midpoint of the humerus with the arm hanging down at the child’s side. Both HL and MUAC were measured to the nearest millimeter using a standard vinyl tape measure.

### Rater reliability

All raters obtaining measurements were required to undergo a quality control assessment prior to their involvement with the study participants. Raters performed each of the study related anthropometric measurements in triplicate on 6 adult volunteers to assess inter- and intra-rater reliability. Intra-rater reliability was required to be less than 5% for each anthropometric measure across all volunteers in order for the individual to qualify as a study rater.

### Data analysis

Data were collected on individual case report forms (CRFs) and verified before entry into an Excel database. Data entry was performed by a single investigator and independently verified by a second study team member against hard copies of the original CRFs. The Mercy method of weight estimation was applied to the quality assured data as previously described [21,23]. In brief, the MUAC and HL measures for each child were rounded up or down to the nearest 1.0 cm. The corresponding fractional weight value for was obtained from the published table and then the fractional weights for MUAC and HL summed to generate an estimated weight for that child. The largest weight value was assigned to children whose humeral length exceeded the upper bound (i.e. the largest value) of the published Mercy method [23]. Data on age and height were similarly applied to four other commonly used weight estimation methods; Advanced Pediatric Life Support (APLS), Australian Resuscitation Council (ARC), Broselow, and Nelson [10-12,15].

The predictive performance of each weight estimation method was evaluated statistically and graphically. Residual error (RE) was calculated by taking the difference of the predicted and actual weights. Percentage error (PE) was calculated by dividing the actual weight into the ME and multiplying by 100. Root mean square error (RMSE) was calculated by taking the square root of the average squared error. Agreement between estimated weight and actual weight was determined by calculating the intraclass correlation coefficient. Bland-Altman plots, with 95% limits of agreement, were constructed to evaluate the agreement between the each weight estimation method and the observed weight. Given that traditional Bland-Altman plots assume that the mean and standard deviation of the differences are constant across the range which is often not the case when weight is examined over a broad range of pediatric ages, the plots constructed for this study were generated from log-transformed data. Finally, to evaluate reliability between raters, the intraclass correlation coefficient (ICC) was determined using a two-way random effects model and an absolute agreement definition. All mathematical and statistical analyses were performed with Microsoft Excel 2003 and SPSS v12.

### Ethics

The study protocol and inform consent document were reviewed and approved by the Ethical Committee of the Faculty of Medicine, Pharmacy and Dentistry of the University of Bamako, and the Institutional Review Board of Children Mercy Hospital in Kansas City, Missouri. Individual, written, informed consent was obtained from a parent or guardian of each subject, prior to screening and enrolment, in addition of the assent from children over the age of 7 years.

## Results

**Figure 1:**
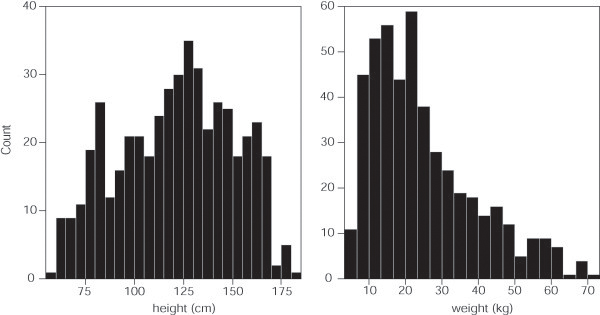
Distribution of pediatric study participants by height and weight.

In total, 473 children were enrolled in this study. Their demographic and anthropometric constitution is detailed in Table 1. Participating children were evenly distributed across gender and age. Expectedly, the population distribution for height was negatively skewed and the distribution for weight positively skewed (Figure 1) resulting in an average body mass index (BMI) and BMI percentile that favored children who were underweight or normal as classified by the Centers for Disease Control (Table 1).

**Table 1:** Demographic and anthropometric characteristics of the children enrolled in the study

**Number of subjects enrolled**	**473**
Male	46.7%
Age (yr)	8.1 ± 4.8
Weight (kg)	25.1 ± 14.5
Height (cm)	120.9 ± 29.5
Humerus (cm)	25.3 ± 6.8
MUAC (cm)	17.8 ± 3.7
BMI (kg/m2)	15.6 ± 2.4
BMI percentile	23.1 ± 23.5
Infant	11.7%
Underweight	21.8%
Normal	64.8%
Overweight	1.3%
Obese	0.4%

All data are provided as mean ± SD unless otherwise indicated.

**Figure 2:**
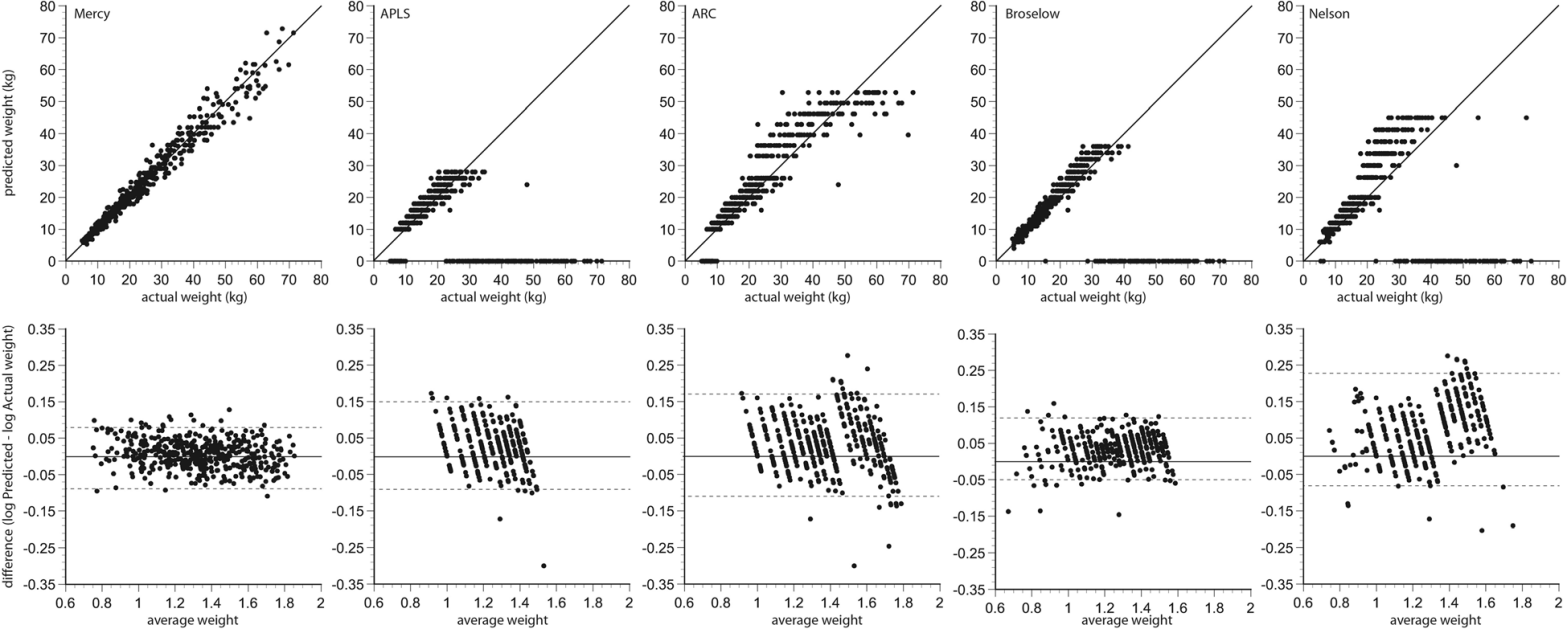
**(Upper) Actual vs. predicted weight for the 5 weight estimation methods.** The solid line represent the line of unity. (Lower) Modified Bland-Altman plots depicting the log-transformed difference between predicted weight and actual weight vs. average log weight. Dashed lines depict the 95% limits of agreements.

**Figure 3:**
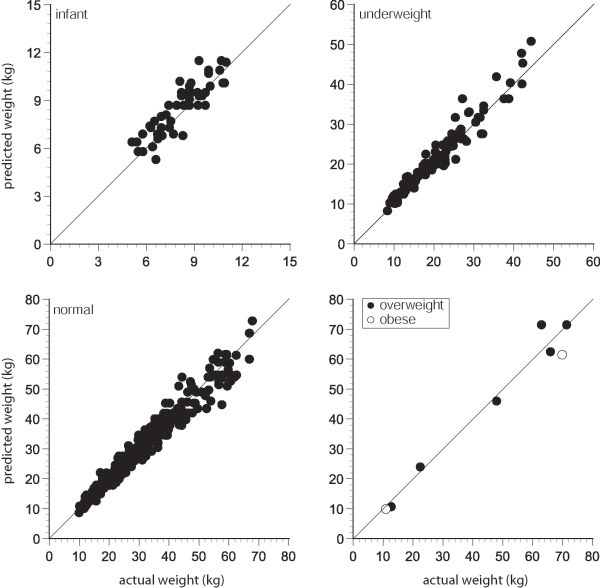
**Actual vs. Mercy predicted weight for children in Ouelessebougou displayed by BMI percentile classification.** The solid line represents the line of unity.

The overall performance of the Mercy method in estimating the weight of children in Ouelessebougou is described in Table 2 and depicted in Figure 2. The Mercy method performed extremely well, demonstrating performance characteristics comparable to those observed for the method when applied to children in the U.S. [21,23]. Moreover, there was no loss in predictive performance when the data were segregated by BMI percentile group (Figure 3). The average absolute error in this study was 0.06 kg which represented a percentage error of 1.6% and the back transformed 95% limits of agreements were 0.85 and 1.21. Collectively, these data suggest a slight overestimation of weight by the Mercy method. Importantly, the method predicted weight within 10% of actual weight for 71.5% of the enrolled children and within 20% of actual for more than 96.7% of the children that were studied [Table 2]. In comparison with the Mercy method, none of the comparator weight estimation methods predicted weight in 100% of the participants enrolled in this study (range: 73-94%, Table 2) because of restrictions in the range of ages or lengths to which the method is applicable. All four methods overestimated weight to a greater extent than was observed for the Mercy method, demonstrated poorer predictive performance statistics with 58-82% of estimated weights within 20% of the children’s actual weight (Table 2), and demonstrated wider limits of agreement (Figure 2).

**Table 2:** Regression parameters and predictive performance of the Mercy method (overall and by rater) and the other comparator weight estimation methods

**Parameter**	**Mercy**					**APLS**	**ARC**	**Broselow**	**Nelson**
	**Overall**	**Rater 1**	**Rater 2**	**Rater 3**	**Rater 4**				
**n=**	**473**	**102**	**200**	**106**	**65**	**345**	**446**	**365**	**369**
RE (kg)	0.06 ± 2.58	0.38 ± 1.69	-0.85 ± 2.59	1.80 ± 2.15	-0.46 ± 2.86	4.36 ± 5.27	1.61 ± 5.48	1.50 ± 2.00	4.10 ± 5.30
	[-12.8,9.7]	[-3.0,8.6]	[-9.0,9.3]	[-3.6,9.7]	[-12.8,5.7]	[-26.8,17.4]	[-30.2,22.4]	[-6.4,8.9]	[-24.8,18.7]
PE (%)	1.6 ± 9.3	1.8 ± 7.9	-1.1 ± 9.2	7.3 ± 8.2	0.4 ± 9.2	23.6 ± 21.4	9.6 ± 18.0	8.2 ± 10.4	19.8 ± 21.6
	[-22.2,34.3]	[-19.2,22.0]	[-19.7,34.3]	[-7.7,30.0]	[-22.2,26.1]	[-38.4,89.9]	[-49.9,89.0]	[-28.6,44.3]	[-37.4,88.5]
RMSE (kg)	2.58	1.73	2.72	2.80	2.88	7.07	5.71	2.49	6.70
ICC	0.992	0.993	0.991	0.989	0.993	0.865	0.956	0.976	0.891
Agreement within:									
10%	71.5%	77.5%	71.5%	67.0%	69.2%	15.0%	40.8%	41.2%	23.5%
20%	96.7	98.0	97.5	93.4	96.9	28.1	68.7	67.4	40.4
30%	99.9	100	99.5	100	100	39.3	82.2	75.1	54.6

All data are presented as mean with (standard deviation) and/or [range] unless otherwise indicated.

n-number of subjects, RE- residual error, PE- percentage error, RMSE- root mean square error, LOA- limits of agreement, ICC- intraclass correlation coefficient.

Three children in this study had an humeral length that exceeded the upper bound of the published Mercy method. In these children, the largest fractional weight for humeral length was assigned and resulted in predicted weights that were within 4.1, 4.5, and 11.5% of actual weight for each child, respectively. By contrast, no child presented with a HL below the smallest bin value of the published method. Similarly, no measures fell beyond the lower or upper bounds of MUAC.

The coefficients of variation for intra-rater variability assessed prior to study initiation were very low averaging 0.11%, 1.36% and 0.67% for height, UAC and HL measures, respectively. Inter-rater reliability, as measured by an ICC of 0.998, was also excellent. Despite the high level of agreement between raters, predictive performance of the Mercy method did appear to demonstrate modest differences between raters (Table 2).

## Discussion and conclusion

Weight estimation tools address a critical medical need in settings where there is no opportunity to directly weigh children. No previously published method provides accurate estimates of weight across a broad range of age, length, stature and ethnicity. By using surrogates for length and girth, the Mercy Method addresses the principal limitations inherent in many of the existing weight estimation strategies and permits application across a broader pediatric population.

In Malian children, the Mercy Method demonstrated goodness-of-fit criteria comparable to those observed for the method when applied to children in the U.S. and superior to those of other commonly used methods [21,23]. The average absolute error observed in this study (0.06 kg) represents slight overestimation of weight by the Mercy Method with comparable performance across all BMI percentile categories. Importantly, the Mercy Method predicted weight within 10% of actual weight for more than 70% of the enrolled children and within 20% of actual for more than 95% of the children that were studied. The performance of the Mercy Method in Malian children comparable to those observed for the method when applied to children in the U.S. and India. This indicated that the Mercy Method could be used for accurate weight estimation without modification in west and sub-Saharan Africa children who were more similar in characteristics and environment to Malian children. Consequently, the Mercy TAPE, a device that applies the Mercy method [24] appears to offer the best option for weight estimation of children.

## Competing interests

Children’s Mercy Hospital (CMH) holds a U.S. patent on the Mercy method. SMA-R is employed by Children’s Mercy Hospital. CMH does not have, never has had, and will not develop an interest in extracting royalties for use of the invention in developing countries or in situations in which royalties could hinder adoption of this medically advantageous device.

The other authors (AD, MLA, BS and MT) declare that they have no competing interests.

## Authors’ contributions

AD, SM-AR conceived, designed and planned the study and analyze the data. Data collection and/supervision was done by AD, MLA, BS, MT and SM-AR. The manuscript was written and reviewed by SM-AR and AD. All authors read and approved the final version.

## Pre-publication history

The pre-publication history for this paper can be accessed here:

http://www.biomedcentral.com/1471-2458/14/270/prepub
